# 4-Hy­droxy-3-[(4-hy­droxy-6-methyl-2-oxo-3,6-dihydro-2*H*-pyran-3-yl)(3-thien­yl)meth­yl]-6-methyl-3,6-dihydro-2*H*-pyran-2-one

**DOI:** 10.1107/S1600536811002716

**Published:** 2011-01-26

**Authors:** Mohammad Asad, Chuan-Wei Oo, Hasnah Osman, Madhukar Hemamalini, Hoong-Kun Fun

**Affiliations:** a School of Chemical Sciences, Universiti Sains Malaysia, 11800 USM, Penang, Malaysia; b X-ray Crystallography Unit, School of Physics, Universiti Sains Malaysia, 11800 USM, Penang, Malaysia

## Abstract

The asymmetric unit of the title compound, C_17_H_14_O_6_S, contains four crystallographically independent mol­ecules in which the pyran­one units are essentially planar, with maximum deviations of 0.016 (2), 0.019 (2), 0.025 (2), 0.014 (2), 0.020 (2), 0.010 (2), 0.003 (2) and 0.012 (2) Å. One of the thio­phene rings is disordered over two positions, with an occupancy ratio of 0.739 (4):0.261 (4). The dihedral angles between the two pyran­one rings in the independent mol­ecules are 59.42 (8), 48.67 (8), 60.62 (9) and 51.60 (8)°. In the crystal, mol­ecules are linked through inter­molecular O—H⋯O and C—H⋯O hydrogen bonds, forming a three-dimensional network.

## Related literature

For general background to the synthesis and activities of polyketide compounds, see: Bentley (1999 ▶); Eckermann *et al.* (2003 ▶); Abe *et al.* (2005 ▶); Fang *et al.* (2010 ▶); Lee *et al.* (2000 ▶); Shyamsunder & Hermann (1999 ▶). For bond-length data, see: Allen *et al.* (1987 ▶). For the stability of the temperature controller used in the data collection, see: Cosier & Glazer (1986 ▶).
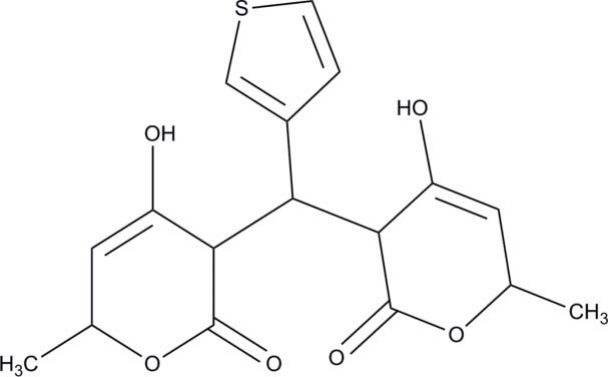


## Experimental

### 

#### Crystal data

C_17_H_14_O_6_S
*M* *_r_* = 346.34
Orthorhombic, 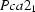

*a* = 30.6120 (18) Å
*b* = 7.9982 (5) Å
*c* = 25.6540 (15) Å
*V* = 6281.1 (7) Å^3^
*Z* = 16
Mo *K*α radiation
μ = 0.24 mm^−1^
*T* = 100 K
0.45 × 0.40 × 0.18 mm

#### Data collection

Bruker APEXII DUO CCD area-detector diffractometer
Absorption correction: multi-scan (*SADABS*; Bruker, 2009 ▶) *T*_min_ = 0.902, *T*_max_ = 0.958
127650 measured reflections
19406 independent reflections
18459 reflections with *I* > 2σ(*I*)
*R*_int_ = 0.034

#### Refinement

*R*[*F* ^2^ > 2σ(*F* ^2^)] = 0.047
*wR*(*F* ^2^) = 0.131
*S* = 1.07
19406 reflections
896 parameters
77 restraints
H-atom parameters constrained
Δρ_max_ = 0.98 e Å^−3^
Δρ_min_ = −0.75 e Å^−3^
Absolute structure: Flack (1983 ▶), 9485 Friedel pairs
Flack parameter: 0.03 (4)

### 

Data collection: *APEX2* (Bruker, 2009 ▶); cell refinement: *SAINT* (Bruker, 2009 ▶); data reduction: *SAINT*; program(s) used to solve structure: *SHELXTL* (Sheldrick, 2008 ▶); program(s) used to refine structure: *SHELXTL*; molecular graphics: *SHELXTL*; software used to prepare material for publication: *SHELXTL* and *PLATON* (Spek, 2009 ▶).

## Supplementary Material

Crystal structure: contains datablocks global, I. DOI: 10.1107/S1600536811002716/rz2545sup1.cif

Structure factors: contains datablocks I. DOI: 10.1107/S1600536811002716/rz2545Isup2.hkl

Additional supplementary materials:  crystallographic information; 3D view; checkCIF report

## Figures and Tables

**Table 1:** Hydrogen-bond geometry (Å, °)

*D*—H⋯*A*	*D*—H	H⋯*A*	*D*⋯*A*	*D*—H⋯*A*
O2*A*—H2*OA*⋯O6*A*	0.82	1.78	2.562 (2)	159
O5*A*—H5*OA*⋯O6*D*	0.82	1.84	2.634 (2)	164
O2*B*—H2*OB*⋯O6*C*^i^	0.82	1.85	2.631 (2)	158
O5*B*—H5*OB*⋯O3*B*	0.82	1.71	2.529 (2)	171
O2*C*—H2*OC*⋯O6*B*^ii^	0.82	1.84	2.663 (2)	175
O5*C*—H5*OC*⋯O3*C*	0.82	1.76	2.549 (2)	163
O2*D*—H2*OD*⋯O3*A*	0.82	1.82	2.6205 (19)	166
O5*D*—H5*OD*⋯O3*D*	0.82	1.81	2.546 (2)	148
C3*A*—H3*AA*⋯O3*C*	0.93	2.45	3.306 (3)	154
C8*A*—H8*AA*⋯O6*D*	0.93	2.38	3.072 (2)	131
C3*B*—H3*BA*⋯O6*A*^iii^	0.93	2.51	3.258 (2)	138
C3*C*—H3*CA*⋯O6*B*^ii^	0.93	2.52	3.193 (3)	130
C11*A*—H11*A*⋯O3*A*	0.98	2.34	2.871 (2)	113
C11*A*—H11*A*⋯O5*A*	0.98	2.30	2.805 (2)	111
C11*B*—H11*B*⋯O2*B*	0.98	2.22	2.768 (2)	114
C11*B*—H11*B*⋯O6*B*	0.98	2.27	2.835 (2)	115
C11*C*—H11*C*⋯O2*C*	0.98	2.35	2.826 (2)	109
C11*C*—H11*C*⋯O6*C*	0.98	2.36	2.875 (2)	112
C11*D*—H11*D*⋯O2*D*	0.98	2.23	2.777 (2)	114
C11*D*—H11*D*⋯O6*D*	0.98	2.27	2.833 (2)	115
C13*A*—H13*A*⋯O4*C*^iv^	0.93	2.39	3.278 (8)	159
C13*B*—H13*C*⋯O1*A*^v^	0.93	2.51	3.311 (3)	145
C13*D*—H13*E*⋯O1*B*^vi^	0.93	2.59	3.366 (2)	142
C14*A*—H14*A*⋯O3*C*^vii^	0.93	2.33	3.183 (4)	152
C14*C*—H14*D*⋯O1*C*^vii^	0.93	2.52	3.377 (2)	154
C16*B*—H16*D*⋯O6*A*^iii^	0.96	2.58	3.382 (3)	141

Symmetry codes: (i) 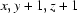; (ii) 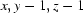; (iii) 
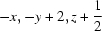
; (iv) 
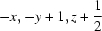
; (v) 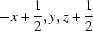; (vi) 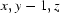; (vii) 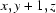.

**Fig. 1.**
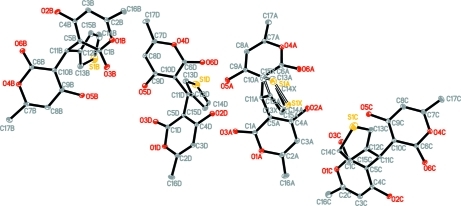
The asymmetric unit of the title compound, showing 30% probability displacement ellipsoids and the atom-numbering scheme. Open bonds represents disorder components [H atoms are omitted for clarity].

**Fig. 2.**
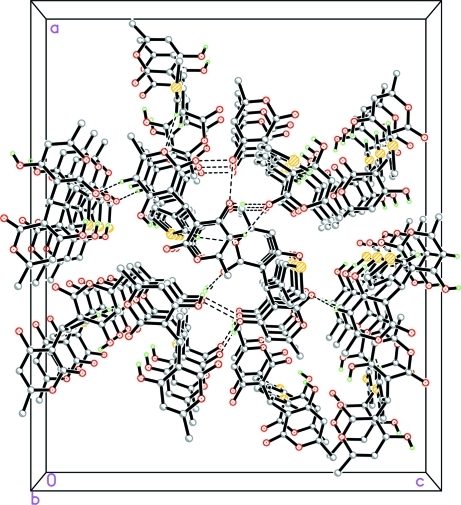
The crystal packing of the title compound, showing the hydrogen-bonded (dashed lines) network.

## supplementary crystallographic information

### Comment

4-Hydroxy-6-methyl-2-pyrone (triacetic acid lactone) is a polyketide (Bentley, 1999) for polyketomethylene compounds CH_3_(-CO–CH_2_-)_n_ and is absorbed in the biosynthesis of  natural products. In recent years, triacetic acid lactone has been found to be the precursor of a phytoalexin in plant Gerbara hybrid (Eckermann *et al.*, 2003; Abe *et al.*, 2005). Polyketides exist as diverse groups in many natural products exhibiting significant physiological effects and their derivatives have been used as anticancer (Fang *et al.*, 2010), anti-HIV (Lee *et al.*, 2000), convulsants and antiepileptics agents (Shyamsunder & Hermann,  1999).

The asymmetric unit of the title compound, consists of four crystallographically independent molecules, (A, B, C & D), as shown in Fig. 1. The bond lengths and angles of molecules A, B, C and D agree with each other and are within normal ranges (Allen *et al.*, 1987). Each molecule consists of two pyranone rings and one thiophene ring. The pyranone units are essentially planar with maximum deviations of 0.016 (2) Å for atom C2A: 0.019 (2) Å for atom C9A (molecule A), 0.025 (2) Å for atom C5B: 0.014 (2) Å for atom C10B (molecule B), 0.020 (2) Å for atom O1C: 0.010 (2) Å for atom C7C (molecule C) and 0.003 (2) Å for atom C10D : 0.012 (2) Å for atom C5D (molecule D). In molecule A, the thiophene ring is disordered over two positions, with occupancy ratio of 0.739 (4):0.261 (4). The dihedral angle between the two pyranone (O1/C1–C5)/(O4/C6–C10) rings are: 59.42 (8)° in molecule A, 48.67 (8)° in molecule B, 60.62 (9)° in molecule C and 51.60 (8)° in molecule D.

In the crystal structure (Fig. 2), the molecules are linked through intermolecular O—H···O and C—H···O hydrogen bonds (Table 1) to form a three-dimensional network.

### Experimental

A mixture of thiophene-3-carboxaldehyde (4.46 mmol, 0.5 g) and 4-hydroxy-6-methylpyrone (8.92 mmol, 1.12 g) in 20 ml of methanol was stirred at room temperature for overnight. The completion of the reaction was monitored by TLC. After the reaction was completed, the crude product that separated, filtered, washed with methanol and dried. The isolated product was further purified by recrystallization from chloroform-methanol (1:1 *v*/*v*) to give the pure title compound in 67% yield.

### Refinement

All the H atoms were positioned geometrically [O–H = 0.82 Å and C–H = 0.93–0.98 Å ] and were refined using a riding model, with *U*_iso_(H) = 1.2 or 1.5 *U*_eq_(C,O). In molecule A, the thiophene ring is disordered over two positions, with occupancy ratio of 0.739 (4):0.261 (4). 9485 Friedel pairs were used to determine the absolute structure. The disordered atoms were subjected to similarity (SAME) and rigid body (SIMU & DELU) restraints. Atoms C14X and C15X were restrained to have the same thermal parameters.

### Crystal data

**Table:**

C_17_H_14_O_6_S	*F*(000) = 2880
*M**_r_* = 346.34	*D*_x_ = 1.465 Mg m^−^^3^
Orthorhombic, *P**c**a*2_1_	Mo *K*α radiation, λ = 0.71073 Å
Hall symbol: P 2c -2ac	Cell parameters from 9836 reflections
*a* = 30.6120 (18) Å	θ = 2.6–30.6°
*b* = 7.9982 (5) Å	µ = 0.24 mm^−^^1^
*c* = 25.6540 (15) Å	*T* = 100 K
*V* = 6281.1 (7) Å^3^	Block, colourless
*Z* = 16	0.45 × 0.40 × 0.18 mm

### Data collection

**Table:**

Bruker APEXII DUO CCD area-detector diffractometer	19406 independent reflections
Radiation source: fine-focus sealed tube	18459 reflections with *I* > 2σ(*I*)
graphite	*R*_int_ = 0.034
φ and ω scans	θ_max_ = 30.7°, θ_min_ = 1.6°
Absorption correction: multi-scan (*SADABS*; Bruker, 2009)	*h* = −43→42
*T*_min_ = 0.902, *T*_max_ = 0.958	*k* = −11→11
127650 measured reflections	*l* = −36→36

### Refinement

**Table:**

Refinement on *F*^2^	Secondary atom site location: difference Fourier map
Least-squares matrix: full	Hydrogen site location: inferred from neighbouring sites
*R*[*F*^2^ > 2σ(*F*^2^)] = 0.047	H-atom parameters constrained
*wR*(*F*^2^) = 0.131	*w* = 1/[σ^2^(*F*_o_^2^) + (0.0845*P*)^2^ + 2.3923*P*] where *P* = (*F*_o_^2^ + 2*F*_c_^2^)/3
*S* = 1.07	(Δ/σ)_max_ = 0.001
19406 reflections	Δρ_max_ = 0.98 e Å^−^^3^
896 parameters	Δρ_min_ = −0.75 e Å^−^^3^
77 restraints	Absolute structure: Flack (1983), 9485 Friedel pairs
Primary atom site location: structure-invariant direct methods	Flack parameter: 0.03 (4)

### Special details

**Table:**

Experimental. The crystal was placed in the cold stream of an Oxford Cryosystems Cobra open-flow nitrogen cryostat (Cosier & Glazer, 1986) operating at 100.0 (1) K.
Geometry. All s.u.'s (except the s.u. in the dihedral angle between two l.s. planes) are estimated using the full covariance matrix. The cell s.u.'s are taken into account individually in the estimation of s.u.'s in distances, angles and torsion angles; correlations between s.u.'s in cell parameters are only used when they are defined by crystal symmetry. An approximate (isotropic) treatment of cell s.u.'s is used for estimating s.u.'s involving l.s. planes.
Refinement. Refinement of F^2^ against ALL reflections. The weighted R-factor wR and goodness of fit S are based on F^2^, conventional R-factors R are based on F, with F set to zero for negative F^2^. The threshold expression of F^2^ > 2σ(F^2^) is used only for calculating R-factors(gt) etc. and is not relevant to the choice of reflections for refinement. R-factors based on F^2^ are statistically about twice as large as those based on F, and R- factors based on ALL data will be even larger.

### Fractional atomic coordinates and isotropic or equivalent isotropic displacement parameters (Å^2^)

**Table:**

	*x*	*y*	*z*	*U*_iso_*/*U*_eq_	Occ. (<1)
O1A	0.18378 (4)	0.51727 (19)	0.33172 (6)	0.0214 (3)	
O2A	0.05929 (5)	0.5288 (2)	0.27713 (6)	0.0226 (3)	
H2OA	0.0416	0.5692	0.2976	0.034*	
O3A	0.16825 (4)	0.65005 (19)	0.40437 (5)	0.0199 (3)	
O4A	−0.02656 (4)	0.46362 (17)	0.40941 (5)	0.0172 (2)	
O5A	0.08386 (4)	0.6121 (2)	0.48443 (5)	0.0214 (3)	
H5OA	0.0818	0.5864	0.5153	0.032*	
O6A	−0.00616 (4)	0.58564 (18)	0.33678 (5)	0.0185 (2)	
C1A	0.15328 (5)	0.5948 (2)	0.36290 (7)	0.0163 (3)	
C2A	0.17312 (7)	0.4506 (3)	0.28440 (8)	0.0227 (4)	
C3A	0.13173 (7)	0.4531 (3)	0.26732 (8)	0.0223 (4)	
H3AA	0.1245	0.4037	0.2356	0.027*	
C4A	0.09871 (6)	0.5321 (2)	0.29810 (7)	0.0179 (3)	
C5A	0.10908 (5)	0.6060 (2)	0.34550 (7)	0.0147 (3)	
C6A	0.00407 (5)	0.5552 (2)	0.38225 (7)	0.0150 (3)	
C7A	−0.02007 (6)	0.4160 (2)	0.45972 (7)	0.0176 (3)	
C8A	0.01651 (6)	0.4597 (2)	0.48527 (7)	0.0173 (3)	
H8AA	0.0210	0.4254	0.5195	0.021*	
C9A	0.04852 (5)	0.5600 (2)	0.45898 (7)	0.0154 (3)	
C10A	0.04336 (5)	0.6051 (2)	0.40742 (7)	0.0140 (3)	
C11A	0.07803 (5)	0.7065 (2)	0.37934 (6)	0.0141 (3)	
H11A	0.0965	0.7503	0.4074	0.017*	
C12A	0.06062 (5)	0.8629 (2)	0.35192 (7)	0.0150 (3)	
S1A	0.02387 (3)	1.14625 (14)	0.33996 (4)	0.0206 (2)	0.739 (4)
C13A	0.0314 (3)	0.9701 (9)	0.3753 (3)	0.0140 (9)	0.739 (4)
H13A	0.0174	0.9482	0.4067	0.017*	0.739 (4)
C14A	0.05913 (14)	1.0821 (6)	0.29190 (15)	0.0208 (7)	0.739 (4)
H14A	0.0656	1.1432	0.2620	0.025*	0.739 (4)
C15A	0.0767 (3)	0.9266 (9)	0.3037 (3)	0.0165 (13)	0.739 (4)
H15A	0.0966	0.8706	0.2826	0.020*	0.739 (4)
S1X	0.05051 (10)	1.1120 (4)	0.28711 (11)	0.0170 (7)*	0.261 (4)
C13X	0.0720 (10)	0.920 (3)	0.3029 (9)	0.019 (5)*	0.261 (4)
H13B	0.0898	0.8595	0.2803	0.023*	0.261 (4)
C14X	0.0226 (5)	1.1125 (19)	0.3455 (6)	0.030 (3)*	0.261 (4)
H14B	0.0030	1.1958	0.3550	0.036*	0.261 (4)
C15X	0.0322 (13)	0.979 (4)	0.3764 (11)	0.030 (3)*	0.261 (4)
H15B	0.0213	0.9653	0.4100	0.036*	0.261 (4)
C16A	0.21206 (8)	0.3842 (3)	0.25676 (10)	0.0325 (5)	
H16A	0.2033	0.3379	0.2238	0.049*	
H16B	0.2255	0.2985	0.2775	0.049*	
H16C	0.2326	0.4730	0.2510	0.049*	
C17A	−0.05696 (7)	0.3152 (3)	0.48050 (9)	0.0264 (4)	
H17A	−0.0519	0.2908	0.5166	0.040*	
H17B	−0.0592	0.2126	0.4613	0.040*	
H17C	−0.0837	0.3772	0.4770	0.040*	
S1B	0.19369 (3)	0.38051 (7)	0.86180 (3)	0.03968 (15)	
O1B	0.11011 (4)	1.01124 (18)	0.75745 (5)	0.0194 (2)	
O2B	0.11192 (5)	1.0506 (2)	0.91526 (5)	0.0222 (3)	
H2OB	0.0913	1.1078	0.9251	0.033*	
O3B	0.17153 (5)	0.8700 (2)	0.75707 (6)	0.0233 (3)	
O4B	0.28401 (4)	1.08993 (19)	0.92247 (5)	0.0206 (3)	
O5B	0.25081 (5)	0.8741 (2)	0.78469 (6)	0.0263 (3)	
H5OB	0.2244	0.8711	0.7791	0.039*	
O6B	0.22134 (5)	1.0353 (2)	0.95991 (6)	0.0256 (3)	
C1B	0.14492 (6)	0.9453 (2)	0.78447 (7)	0.0169 (3)	
C2B	0.07627 (6)	1.0898 (2)	0.78185 (8)	0.0183 (3)	
C3B	0.07591 (6)	1.1069 (2)	0.83392 (8)	0.0189 (3)	
H3BA	0.0527	1.1599	0.8505	0.023*	
C4B	0.11181 (6)	1.0424 (2)	0.86360 (7)	0.0163 (3)	
C5B	0.14690 (5)	0.9671 (2)	0.83935 (7)	0.0156 (3)	
C6B	0.24240 (6)	1.0255 (2)	0.91906 (7)	0.0177 (3)	
C7B	0.31275 (6)	1.0808 (3)	0.88188 (8)	0.0192 (3)	
C8B	0.30122 (6)	1.0103 (3)	0.83702 (8)	0.0213 (3)	
H8BA	0.3212	1.0043	0.8098	0.026*	
C9B	0.25807 (6)	0.9434 (2)	0.83045 (7)	0.0183 (3)	
C10B	0.22801 (6)	0.9527 (2)	0.87098 (7)	0.0151 (3)	
C11B	0.18205 (5)	0.8820 (2)	0.87199 (7)	0.0144 (3)	
H11B	0.1725	0.9007	0.9080	0.017*	
C12B	0.17879 (6)	0.6940 (2)	0.86535 (7)	0.0184 (3)	
C13B	0.21236 (7)	0.5834 (3)	0.86135 (9)	0.0257 (4)	
H13C	0.2416	0.6135	0.8587	0.031*	
C14B	0.14072 (10)	0.4434 (3)	0.86795 (11)	0.0367 (5)	
H14C	0.1171	0.3707	0.8701	0.044*	
C15B	0.13725 (7)	0.6125 (3)	0.86926 (8)	0.0244 (4)	
H15C	0.1108	0.6691	0.8724	0.029*	
C16B	0.04203 (6)	1.1457 (3)	0.74444 (8)	0.0220 (3)	
H16D	0.0189	1.1999	0.7632	0.033*	
H16E	0.0306	1.0505	0.7262	0.033*	
H16F	0.0546	1.2226	0.7199	0.033*	
C17B	0.35575 (6)	1.1554 (3)	0.89567 (9)	0.0249 (4)	
H17D	0.3718	1.1793	0.8644	0.037*	
H17E	0.3720	1.0780	0.9167	0.037*	
H17F	0.3512	1.2570	0.9148	0.037*	
S1C	0.04116 (2)	0.69647 (9)	0.14816 (3)	0.03862 (14)	
O1C	0.16141 (5)	0.0454 (2)	0.16312 (6)	0.0234 (3)	
O2C	0.15261 (5)	0.1631 (2)	0.00950 (5)	0.0244 (3)	
H2OC	0.1745	0.1291	−0.0056	0.037*	
O3C	0.09952 (5)	0.1718 (2)	0.18105 (5)	0.0224 (3)	
O4C	−0.00688 (4)	0.07788 (18)	−0.00124 (5)	0.0190 (2)	
O5C	0.02024 (5)	0.1421 (2)	0.15213 (6)	0.0279 (3)	
H5OC	0.0456	0.1691	0.1578	0.042*	
O6C	0.05246 (4)	0.20544 (18)	−0.02862 (5)	0.0183 (2)	
C1C	0.12592 (6)	0.1351 (2)	0.14610 (7)	0.0185 (3)	
C2C	0.19240 (7)	−0.0128 (3)	0.12962 (9)	0.0251 (4)	
C3C	0.19004 (6)	0.0210 (3)	0.07856 (8)	0.0212 (3)	
H3CA	0.2110	−0.0204	0.0557	0.025*	
C4C	0.15481 (6)	0.1216 (2)	0.05961 (7)	0.0178 (3)	
C5C	0.12247 (6)	0.1784 (2)	0.09278 (7)	0.0156 (3)	
C6C	0.03171 (5)	0.1608 (2)	0.01014 (7)	0.0151 (3)	
C7C	−0.03533 (6)	0.0280 (3)	0.03633 (8)	0.0226 (4)	
C8C	−0.02594 (7)	0.0511 (3)	0.08700 (8)	0.0260 (4)	
H8CA	−0.0454	0.0161	0.1126	0.031*	
C9C	0.01399 (6)	0.1293 (3)	0.10114 (8)	0.0211 (3)	
C10C	0.04287 (6)	0.1874 (2)	0.06350 (7)	0.0163 (3)	
C11C	0.08471 (5)	0.2852 (2)	0.07366 (6)	0.0152 (3)	
H11C	0.0939	0.3255	0.0393	0.018*	
C12C	0.07744 (6)	0.4430 (2)	0.10582 (7)	0.0176 (3)	
C13C	0.03707 (7)	0.5141 (3)	0.11578 (8)	0.0226 (4)	
H13D	0.0107	0.4665	0.1057	0.027*	
C14C	0.09725 (5)	0.7038 (2)	0.15190 (6)	0.0129 (3)	
H14D	0.1146	0.7869	0.1665	0.015*	
C15C	0.11181 (7)	0.5416 (2)	0.12557 (8)	0.0217 (3)	
H15D	0.1410	0.5105	0.1228	0.026*	
C16C	0.22694 (9)	−0.1091 (4)	0.15704 (11)	0.0401 (6)	
H16G	0.2469	−0.1551	0.1319	0.060*	
H16H	0.2138	−0.1981	0.1766	0.060*	
H16I	0.2425	−0.0364	0.1803	0.060*	
C17C	−0.07542 (7)	−0.0479 (3)	0.01396 (10)	0.0301 (4)	
H17G	−0.0925	−0.0970	0.0414	0.045*	
H17H	−0.0674	−0.1327	−0.0108	0.045*	
H17I	−0.0923	0.0370	−0.0032	0.045*	
S1D	0.15685 (2)	−0.16664 (8)	0.62643 (3)	0.04059 (15)	
O1D	0.28328 (4)	0.4540 (2)	0.56940 (5)	0.0209 (3)	
O2D	0.16743 (4)	0.4828 (2)	0.49220 (5)	0.0209 (3)	
H2OD	0.1714	0.5255	0.4635	0.031*	
O3D	0.25285 (5)	0.32111 (19)	0.63531 (5)	0.0213 (3)	
O4D	0.07404 (4)	0.54489 (18)	0.65873 (5)	0.0183 (2)	
O5D	0.19204 (5)	0.3598 (2)	0.70194 (6)	0.0255 (3)	
H5OD	0.2057	0.3158	0.6781	0.038*	
O6D	0.08083 (5)	0.4709 (2)	0.57712 (6)	0.0234 (3)	
C1D	0.24636 (6)	0.3866 (2)	0.59289 (7)	0.0169 (3)	
C2D	0.28167 (6)	0.5303 (2)	0.52211 (7)	0.0189 (3)	
C3D	0.24399 (6)	0.5423 (2)	0.49556 (7)	0.0176 (3)	
H3DA	0.2432	0.5944	0.4632	0.021*	
C4D	0.20522 (5)	0.4740 (2)	0.51777 (7)	0.0153 (3)	
C5D	0.20566 (5)	0.3995 (2)	0.56627 (7)	0.0146 (3)	
C6D	0.09888 (6)	0.4738 (2)	0.62020 (7)	0.0159 (3)	
C7D	0.08810 (6)	0.5552 (2)	0.70891 (7)	0.0185 (3)	
C8D	0.12725 (6)	0.4953 (2)	0.72266 (7)	0.0188 (3)	
H8DA	0.1366	0.5029	0.7571	0.023*	
C9D	0.15475 (6)	0.4190 (2)	0.68389 (7)	0.0172 (3)	
C10D	0.14102 (5)	0.4083 (2)	0.63250 (7)	0.0143 (3)	
C11D	0.16413 (5)	0.3204 (2)	0.58779 (7)	0.0142 (3)	
H11D	0.1434	0.3296	0.5589	0.017*	
C12D	0.16894 (6)	0.1322 (2)	0.59551 (7)	0.0159 (3)	
C13D	0.15254 (7)	0.0422 (2)	0.63678 (8)	0.0222 (4)	
H13E	0.1406	0.0898	0.6666	0.027*	
C14D	0.17998 (6)	−0.1565 (3)	0.56986 (8)	0.0251 (4)	
H14E	0.1884	−0.2472	0.5495	0.030*	
C15D	0.18562 (7)	0.0288 (3)	0.55539 (9)	0.0243 (4)	
H15E	0.1982	0.0674	0.5247	0.029*	
C16D	0.32537 (6)	0.5914 (3)	0.50485 (9)	0.0277 (4)	
H16J	0.3221	0.6581	0.4740	0.042*	
H16K	0.3439	0.4977	0.4975	0.042*	
H16L	0.3382	0.6579	0.5320	0.042*	
C17D	0.05521 (7)	0.6371 (3)	0.74311 (9)	0.0271 (4)	
H17J	0.0666	0.6457	0.7779	0.041*	
H17K	0.0289	0.5715	0.7435	0.041*	
H17L	0.0488	0.7468	0.7300	0.041*	

### Atomic displacement parameters (Å^2^)

**Table:**

	*U*^11^	*U*^22^	*U*^33^	*U*^12^	*U*^13^	*U*^23^
O1A	0.0141 (6)	0.0307 (7)	0.0192 (6)	0.0031 (5)	0.0023 (4)	0.0010 (5)
O2A	0.0194 (6)	0.0321 (8)	0.0165 (6)	−0.0001 (5)	−0.0031 (5)	−0.0071 (5)
O3A	0.0146 (5)	0.0294 (7)	0.0158 (6)	−0.0018 (5)	−0.0019 (4)	0.0046 (5)
O4A	0.0145 (5)	0.0206 (6)	0.0163 (6)	−0.0012 (4)	−0.0002 (4)	−0.0009 (5)
O5A	0.0151 (6)	0.0336 (8)	0.0156 (6)	−0.0040 (5)	−0.0051 (4)	0.0046 (5)
O6A	0.0181 (6)	0.0216 (6)	0.0159 (6)	0.0007 (5)	−0.0046 (4)	0.0004 (5)
C1A	0.0140 (7)	0.0195 (8)	0.0155 (7)	0.0021 (6)	0.0016 (6)	0.0033 (6)
C2A	0.0243 (9)	0.0242 (9)	0.0194 (8)	0.0036 (7)	0.0057 (7)	−0.0015 (7)
C3A	0.0245 (9)	0.0242 (9)	0.0181 (8)	−0.0004 (7)	0.0023 (7)	−0.0058 (7)
C4A	0.0184 (8)	0.0198 (8)	0.0154 (7)	−0.0005 (6)	−0.0011 (6)	−0.0019 (6)
C5A	0.0122 (7)	0.0189 (7)	0.0131 (7)	−0.0003 (5)	−0.0005 (5)	−0.0003 (6)
C6A	0.0130 (7)	0.0147 (7)	0.0173 (7)	0.0005 (5)	−0.0009 (5)	−0.0026 (6)
C7A	0.0164 (7)	0.0185 (8)	0.0179 (8)	0.0005 (6)	0.0017 (6)	0.0006 (6)
C8A	0.0150 (7)	0.0208 (8)	0.0160 (7)	0.0024 (6)	0.0009 (6)	0.0028 (6)
C9A	0.0124 (7)	0.0184 (7)	0.0154 (7)	0.0020 (6)	−0.0012 (5)	−0.0004 (6)
C10A	0.0139 (7)	0.0145 (7)	0.0134 (7)	0.0013 (5)	−0.0019 (5)	−0.0013 (6)
C11A	0.0133 (6)	0.0158 (7)	0.0131 (7)	−0.0009 (5)	−0.0006 (5)	−0.0015 (5)
C12A	0.0128 (7)	0.0162 (7)	0.0158 (7)	−0.0020 (5)	−0.0012 (5)	−0.0011 (6)
S1A	0.0233 (4)	0.0175 (4)	0.0211 (4)	0.0025 (3)	0.0017 (2)	0.0036 (3)
C13A	0.0153 (12)	0.0140 (13)	0.0126 (12)	−0.0007 (11)	0.0041 (8)	0.0014 (9)
C14A	0.0179 (15)	0.0266 (17)	0.0179 (14)	−0.0012 (13)	0.0035 (11)	−0.0014 (12)
C15A	0.018 (2)	0.0189 (19)	0.0126 (17)	−0.0041 (15)	0.0019 (13)	−0.0014 (9)
C16A	0.0251 (10)	0.0414 (12)	0.0309 (11)	0.0083 (9)	0.0109 (8)	−0.0038 (9)
C17A	0.0229 (9)	0.0302 (10)	0.0262 (10)	−0.0080 (7)	0.0047 (7)	0.0018 (8)
S1B	0.0560 (4)	0.0189 (2)	0.0442 (3)	0.0073 (2)	−0.0067 (3)	0.0008 (2)
O1B	0.0167 (6)	0.0249 (6)	0.0164 (6)	0.0035 (5)	−0.0011 (5)	0.0012 (5)
O2B	0.0207 (6)	0.0309 (7)	0.0150 (6)	0.0074 (5)	0.0035 (5)	−0.0027 (5)
O3B	0.0223 (6)	0.0315 (7)	0.0161 (6)	0.0076 (6)	0.0013 (5)	−0.0037 (5)
O4B	0.0152 (6)	0.0290 (7)	0.0176 (6)	0.0013 (5)	0.0009 (5)	−0.0035 (5)
O5B	0.0180 (6)	0.0447 (9)	0.0162 (6)	−0.0013 (6)	0.0039 (5)	−0.0062 (6)
O6B	0.0174 (6)	0.0438 (9)	0.0156 (6)	0.0020 (6)	0.0011 (5)	−0.0065 (6)
C1B	0.0158 (7)	0.0197 (8)	0.0151 (7)	0.0000 (6)	−0.0004 (6)	0.0005 (6)
C2B	0.0132 (7)	0.0180 (8)	0.0239 (8)	−0.0001 (6)	−0.0020 (6)	−0.0010 (7)
C3B	0.0128 (7)	0.0194 (8)	0.0246 (9)	0.0015 (6)	−0.0014 (6)	−0.0023 (7)
C4B	0.0144 (7)	0.0171 (7)	0.0173 (7)	−0.0003 (6)	0.0008 (6)	−0.0023 (6)
C5B	0.0143 (7)	0.0167 (7)	0.0157 (7)	0.0016 (5)	0.0011 (6)	0.0004 (6)
C6B	0.0140 (7)	0.0226 (8)	0.0165 (7)	0.0038 (6)	−0.0001 (6)	−0.0019 (6)
C7B	0.0118 (7)	0.0234 (9)	0.0225 (9)	0.0025 (6)	0.0026 (6)	0.0031 (7)
C8B	0.0148 (7)	0.0269 (9)	0.0223 (8)	0.0007 (6)	0.0037 (6)	0.0024 (7)
C9B	0.0168 (8)	0.0239 (8)	0.0143 (7)	0.0011 (6)	0.0018 (6)	0.0021 (6)
C10B	0.0144 (7)	0.0182 (7)	0.0128 (7)	0.0020 (6)	0.0022 (5)	0.0004 (6)
C11B	0.0126 (7)	0.0175 (7)	0.0132 (7)	0.0011 (5)	0.0018 (5)	0.0006 (6)
C12B	0.0224 (8)	0.0188 (8)	0.0139 (7)	0.0018 (6)	−0.0013 (6)	0.0017 (6)
C13B	0.0312 (10)	0.0215 (9)	0.0243 (9)	0.0061 (7)	0.0000 (8)	−0.0009 (7)
C14B	0.0492 (14)	0.0251 (11)	0.0356 (12)	−0.0121 (10)	−0.0113 (10)	0.0059 (9)
C15B	0.0281 (9)	0.0239 (9)	0.0213 (9)	−0.0042 (7)	−0.0039 (7)	0.0029 (7)
C16B	0.0175 (8)	0.0237 (9)	0.0249 (9)	0.0021 (6)	−0.0066 (6)	0.0001 (7)
C17B	0.0167 (8)	0.0298 (10)	0.0281 (10)	−0.0006 (7)	−0.0005 (7)	−0.0016 (8)
S1C	0.0457 (3)	0.0341 (3)	0.0361 (3)	0.0085 (2)	0.0047 (3)	−0.0012 (2)
O1C	0.0206 (6)	0.0336 (8)	0.0162 (6)	−0.0011 (5)	−0.0025 (5)	0.0029 (5)
O2C	0.0191 (6)	0.0420 (8)	0.0120 (6)	0.0074 (6)	0.0029 (4)	−0.0020 (6)
O3C	0.0225 (6)	0.0338 (8)	0.0108 (6)	−0.0047 (5)	0.0028 (5)	−0.0007 (5)
O4C	0.0161 (6)	0.0234 (6)	0.0175 (6)	−0.0023 (5)	−0.0028 (5)	0.0000 (5)
O5C	0.0217 (7)	0.0480 (10)	0.0140 (6)	−0.0105 (6)	0.0011 (5)	0.0054 (6)
O6C	0.0173 (6)	0.0242 (6)	0.0133 (5)	0.0036 (5)	0.0023 (4)	−0.0013 (5)
C1C	0.0174 (7)	0.0221 (8)	0.0160 (8)	−0.0049 (6)	−0.0012 (6)	−0.0025 (6)
C2C	0.0212 (8)	0.0306 (10)	0.0234 (9)	0.0014 (7)	−0.0021 (7)	0.0010 (8)
C3C	0.0164 (8)	0.0257 (9)	0.0216 (9)	0.0029 (6)	0.0002 (6)	−0.0030 (7)
C4C	0.0170 (7)	0.0226 (8)	0.0139 (7)	−0.0016 (6)	0.0000 (5)	−0.0038 (6)
C5C	0.0142 (7)	0.0202 (8)	0.0126 (7)	−0.0021 (6)	0.0015 (5)	−0.0028 (6)
C6C	0.0121 (7)	0.0179 (7)	0.0155 (7)	0.0025 (5)	−0.0014 (5)	−0.0015 (6)
C7C	0.0170 (8)	0.0264 (9)	0.0244 (9)	−0.0049 (7)	−0.0019 (7)	0.0047 (7)
C8C	0.0179 (8)	0.0368 (11)	0.0233 (9)	−0.0100 (7)	−0.0018 (7)	0.0087 (8)
C9C	0.0176 (8)	0.0290 (9)	0.0166 (8)	−0.0056 (7)	0.0000 (6)	0.0027 (7)
C10C	0.0143 (7)	0.0217 (8)	0.0130 (7)	−0.0020 (6)	0.0004 (5)	−0.0008 (6)
C11C	0.0153 (7)	0.0191 (7)	0.0110 (7)	−0.0020 (6)	0.0029 (5)	−0.0007 (6)
C12C	0.0205 (8)	0.0195 (8)	0.0129 (7)	0.0003 (6)	0.0043 (6)	0.0008 (6)
C13C	0.0227 (9)	0.0269 (9)	0.0181 (8)	0.0042 (7)	0.0031 (6)	−0.0025 (7)
C14C	0.0111 (6)	0.0179 (7)	0.0097 (6)	−0.0024 (5)	0.0043 (5)	0.0022 (5)
C15C	0.0238 (9)	0.0197 (8)	0.0215 (9)	−0.0039 (7)	0.0031 (7)	−0.0008 (7)
C16C	0.0318 (11)	0.0570 (17)	0.0314 (12)	0.0121 (11)	−0.0031 (9)	0.0095 (11)
C17C	0.0227 (9)	0.0368 (12)	0.0309 (11)	−0.0097 (8)	−0.0070 (8)	0.0044 (9)
S1D	0.0407 (3)	0.0269 (3)	0.0542 (4)	−0.0050 (2)	0.0047 (3)	0.0048 (3)
O1D	0.0129 (6)	0.0329 (7)	0.0167 (6)	−0.0001 (5)	−0.0005 (4)	0.0073 (5)
O2D	0.0153 (6)	0.0318 (7)	0.0155 (6)	0.0000 (5)	−0.0027 (4)	0.0048 (5)
O3D	0.0167 (6)	0.0303 (7)	0.0169 (6)	0.0019 (5)	−0.0009 (5)	0.0066 (5)
O4D	0.0150 (5)	0.0243 (6)	0.0156 (6)	0.0008 (5)	0.0028 (4)	0.0033 (5)
O5D	0.0163 (6)	0.0440 (9)	0.0163 (6)	0.0082 (6)	−0.0026 (5)	−0.0002 (6)
O6D	0.0165 (6)	0.0381 (8)	0.0155 (6)	0.0033 (5)	−0.0008 (5)	0.0041 (6)
C1D	0.0134 (7)	0.0196 (8)	0.0176 (7)	0.0018 (6)	0.0016 (6)	0.0013 (6)
C2D	0.0144 (7)	0.0249 (9)	0.0175 (8)	−0.0011 (6)	0.0014 (6)	0.0047 (7)
C3D	0.0156 (7)	0.0217 (8)	0.0155 (7)	0.0027 (6)	0.0011 (6)	0.0038 (6)
C4D	0.0140 (7)	0.0176 (7)	0.0142 (7)	0.0017 (5)	−0.0004 (5)	−0.0020 (6)
C5D	0.0125 (7)	0.0160 (7)	0.0154 (7)	0.0009 (5)	0.0012 (5)	−0.0004 (6)
C6D	0.0142 (7)	0.0184 (8)	0.0151 (7)	−0.0029 (6)	0.0019 (5)	0.0023 (6)
C7D	0.0201 (8)	0.0187 (8)	0.0168 (8)	−0.0016 (6)	0.0042 (6)	0.0005 (6)
C8D	0.0192 (8)	0.0248 (8)	0.0125 (7)	−0.0006 (6)	0.0007 (6)	−0.0037 (6)
C9D	0.0158 (7)	0.0203 (8)	0.0156 (7)	0.0003 (6)	−0.0012 (5)	−0.0004 (6)
C10D	0.0131 (7)	0.0154 (7)	0.0145 (7)	−0.0011 (5)	0.0009 (5)	−0.0012 (6)
C11D	0.0134 (7)	0.0164 (7)	0.0128 (7)	−0.0001 (5)	−0.0001 (5)	−0.0004 (5)
C12D	0.0147 (7)	0.0165 (7)	0.0164 (7)	−0.0016 (6)	−0.0006 (6)	−0.0007 (6)
C13D	0.0256 (9)	0.0159 (8)	0.0250 (9)	−0.0012 (6)	0.0042 (7)	0.0013 (7)
C14D	0.0196 (8)	0.0302 (10)	0.0254 (9)	0.0037 (7)	−0.0001 (7)	−0.0237 (8)
C15D	0.0266 (9)	0.0220 (9)	0.0243 (9)	0.0016 (7)	0.0047 (7)	−0.0064 (7)
C16D	0.0159 (8)	0.0420 (12)	0.0250 (10)	−0.0052 (8)	0.0031 (7)	0.0112 (9)
C17D	0.0255 (9)	0.0275 (9)	0.0283 (10)	0.0058 (8)	0.0108 (7)	−0.0027 (8)

### Geometric parameters (Å, °)

**Table:**

O1A—C2A	1.365 (3)	C16B—H16F	0.9600
O1A—C1A	1.377 (2)	C17B—H17D	0.9600
O2A—C4A	1.321 (2)	C17B—H17E	0.9600
O2A—H2OA	0.8200	C17B—H17F	0.9600
O3A—C1A	1.240 (2)	S1C—C13C	1.683 (2)
O4A—C7A	1.360 (2)	S1C—C14C	1.7207 (17)
O4A—C6A	1.379 (2)	O1C—C2C	1.362 (3)
O5A—C9A	1.330 (2)	O1C—C1C	1.373 (2)
O5A—H5OA	0.8200	O2C—C4C	1.329 (2)
O6A—C6A	1.232 (2)	O2C—H2OC	0.8200
C1A—C5A	1.428 (2)	O3C—C1C	1.242 (2)
C2A—C3A	1.341 (3)	O4C—C7C	1.359 (2)
C2A—C16A	1.486 (3)	O4C—C6C	1.386 (2)
C3A—C4A	1.430 (3)	O5C—C9C	1.326 (2)
C3A—H3AA	0.9300	O5C—H5OC	0.8200
C4A—C5A	1.389 (2)	O6C—C6C	1.233 (2)
C5A—C11A	1.518 (2)	C1C—C5C	1.415 (2)
C6A—C10A	1.422 (2)	C2C—C3C	1.339 (3)
C7A—C8A	1.344 (2)	C2C—C16C	1.486 (3)
C7A—C17A	1.486 (3)	C3C—C4C	1.431 (3)
C8A—C9A	1.435 (2)	C3C—H3CA	0.9300
C8A—H8AA	0.9300	C4C—C5C	1.382 (2)
C9A—C10A	1.380 (2)	C5C—C11C	1.519 (2)
C10A—C11A	1.518 (2)	C6C—C10C	1.427 (2)
C11A—C12A	1.531 (2)	C7C—C8C	1.344 (3)
C11A—H11A	0.9800	C7C—C17C	1.485 (3)
C12A—C13A	1.376 (5)	C8C—C9C	1.420 (3)
C12A—C13X	1.383 (18)	C8C—H8CA	0.9300
C12A—C15X	1.418 (19)	C9C—C10C	1.389 (2)
C12A—C15A	1.426 (6)	C10C—C11C	1.523 (2)
S1A—C13A	1.691 (6)	C11C—C12C	1.525 (3)
S1A—C14A	1.717 (4)	C11C—H11C	0.9800
C13A—H13A	0.9300	C12C—C13C	1.384 (3)
C14A—C15A	1.387 (9)	C12C—C15C	1.409 (3)
C14A—H14A	0.9300	C13C—H13D	0.9300
C15A—H15A	0.9300	C14C—C15C	1.529 (3)
S1X—C13X	1.720 (18)	C14C—H14D	0.9300
S1X—C14X	1.725 (13)	C15C—H15D	0.9300
C13X—H13B	0.9300	C16C—H16G	0.9600
C14X—C15X	1.36 (2)	C16C—H16H	0.9600
C14X—H14B	0.9300	C16C—H16I	0.9600
C15X—H15B	0.9300	C17C—H17G	0.9600
C16A—H16A	0.9600	C17C—H17H	0.9600
C16A—H16B	0.9600	C17C—H17I	0.9600
C16A—H16C	0.9600	S1D—C14D	1.617 (2)
C17A—H17A	0.9600	S1D—C13D	1.696 (2)
C17A—H17B	0.9600	O1D—C2D	1.359 (2)
C17A—H17C	0.9600	O1D—C1D	1.390 (2)
S1B—C14B	1.705 (3)	O2D—C4D	1.332 (2)
S1B—C13B	1.720 (2)	O2D—H2OD	0.8200
O1B—C2B	1.364 (2)	O3D—C1D	1.224 (2)
O1B—C1B	1.376 (2)	O4D—C7D	1.360 (2)
O2B—C4B	1.327 (2)	O4D—C6D	1.370 (2)
O2B—H2OB	0.8200	O5D—C9D	1.320 (2)
O3B—C1B	1.233 (2)	O5D—H5OD	0.8200
O4B—C7B	1.365 (2)	O6D—C6D	1.236 (2)
O4B—C6B	1.377 (2)	C1D—C5D	1.424 (2)
O5B—C9B	1.317 (2)	C2D—C3D	1.343 (2)
O5B—H5OB	0.8200	C2D—C16D	1.492 (3)
O6B—C6B	1.233 (2)	C3D—C4D	1.425 (2)
C1B—C5B	1.420 (2)	C3D—H3DA	0.9300
C2B—C3B	1.343 (3)	C4D—C5D	1.379 (2)
C2B—C16B	1.490 (3)	C5D—C11D	1.524 (2)
C3B—C4B	1.433 (2)	C6D—C10D	1.428 (2)
C3B—H3BA	0.9300	C7D—C8D	1.338 (3)
C4B—C5B	1.380 (2)	C7D—C17D	1.487 (3)
C5B—C11B	1.524 (2)	C8D—C9D	1.439 (2)
C6B—C10B	1.433 (2)	C8D—H8DA	0.9300
C7B—C8B	1.329 (3)	C9D—C10D	1.386 (2)
C7B—C17B	1.488 (3)	C10D—C11D	1.520 (2)
C8B—C9B	1.435 (3)	C11D—C12D	1.525 (2)
C8B—H8BA	0.9300	C11D—H11D	0.9800
C9B—C10B	1.391 (2)	C12D—C13D	1.375 (3)
C10B—C11B	1.516 (2)	C12D—C15D	1.416 (3)
C11B—C12B	1.516 (3)	C13D—H13E	0.9300
C11B—H11B	0.9800	C14D—C15D	1.537 (3)
C12B—C13B	1.360 (3)	C14D—H14E	0.9300
C12B—C15B	1.433 (3)	C15D—H15E	0.9300
C13B—H13C	0.9300	C16D—H16J	0.9600
C14B—C15B	1.357 (3)	C16D—H16K	0.9600
C14B—H14C	0.9300	C16D—H16L	0.9600
C15B—H15C	0.9300	C17D—H17J	0.9600
C16B—H16D	0.9600	C17D—H17K	0.9600
C16B—H16E	0.9600	C17D—H17L	0.9600
			
C2A—O1A—C1A	122.01 (15)	H16D—C16B—H16F	109.5
C4A—O2A—H2OA	109.5	H16E—C16B—H16F	109.5
C7A—O4A—C6A	121.93 (14)	C7B—C17B—H17D	109.5
C9A—O5A—H5OA	109.5	C7B—C17B—H17E	109.5
O3A—C1A—O1A	114.10 (15)	H17D—C17B—H17E	109.5
O3A—C1A—C5A	126.61 (17)	C7B—C17B—H17F	109.5
O1A—C1A—C5A	119.29 (16)	H17D—C17B—H17F	109.5
C3A—C2A—O1A	120.71 (17)	H17E—C17B—H17F	109.5
C3A—C2A—C16A	127.4 (2)	C13C—S1C—C14C	97.54 (10)
O1A—C2A—C16A	111.85 (19)	C2C—O1C—C1C	121.92 (16)
C2A—C3A—C4A	119.62 (18)	C4C—O2C—H2OC	109.5
C2A—C3A—H3AA	120.2	C7C—O4C—C6C	122.49 (15)
C4A—C3A—H3AA	120.2	C9C—O5C—H5OC	109.5
O2A—C4A—C5A	125.01 (17)	O3C—C1C—O1C	114.12 (17)
O2A—C4A—C3A	114.33 (16)	O3C—C1C—C5C	126.25 (18)
C5A—C4A—C3A	120.65 (17)	O1C—C1C—C5C	119.62 (16)
C4A—C5A—C1A	117.64 (16)	C3C—C2C—O1C	120.73 (19)
C4A—C5A—C11A	125.67 (15)	C3C—C2C—C16C	127.3 (2)
C1A—C5A—C11A	116.60 (15)	O1C—C2C—C16C	111.95 (19)
O6A—C6A—O4A	114.24 (15)	C2C—C3C—C4C	119.11 (18)
O6A—C6A—C10A	126.10 (17)	C2C—C3C—H3CA	120.4
O4A—C6A—C10A	119.66 (16)	C4C—C3C—H3CA	120.4
C8A—C7A—O4A	120.78 (16)	O2C—C4C—C5C	118.50 (17)
C8A—C7A—C17A	126.81 (18)	O2C—C4C—C3C	120.47 (17)
O4A—C7A—C17A	112.41 (16)	C5C—C4C—C3C	121.02 (17)
C7A—C8A—C9A	119.02 (16)	C4C—C5C—C1C	117.47 (17)
C7A—C8A—H8AA	120.5	C4C—C5C—C11C	122.07 (15)
C9A—C8A—H8AA	120.5	C1C—C5C—C11C	120.45 (15)
O5A—C9A—C10A	118.76 (16)	O6C—C6C—O4C	114.06 (15)
O5A—C9A—C8A	119.99 (16)	O6C—C6C—C10C	127.39 (17)
C10A—C9A—C8A	121.25 (16)	O4C—C6C—C10C	118.55 (15)
C9A—C10A—C6A	117.27 (16)	C8C—C7C—O4C	120.58 (17)
C9A—C10A—C11A	120.98 (15)	C8C—C7C—C17C	127.36 (19)
C6A—C10A—C11A	121.74 (15)	O4C—C7C—C17C	112.06 (18)
C10A—C11A—C5A	115.24 (14)	C7C—C8C—C9C	119.44 (18)
C10A—C11A—C12A	114.27 (14)	C7C—C8C—H8CA	120.3
C5A—C11A—C12A	112.84 (14)	C9C—C8C—H8CA	120.3
C10A—C11A—H11A	104.3	O5C—C9C—C10C	124.63 (17)
C5A—C11A—H11A	104.3	O5C—C9C—C8C	114.20 (17)
C12A—C11A—H11A	104.3	C10C—C9C—C8C	121.16 (18)
C13A—C12A—C13X	110.8 (10)	C9C—C10C—C6C	117.68 (16)
C13A—C12A—C15X	3(2)	C9C—C10C—C11C	126.00 (16)
C13X—C12A—C15X	110.0 (9)	C6C—C10C—C11C	116.25 (15)
C13A—C12A—C15A	112.3 (3)	C5C—C11C—C10C	114.01 (15)
C13X—C12A—C15A	6.0 (19)	C5C—C11C—C12C	113.69 (14)
C15X—C12A—C15A	111.2 (11)	C10C—C11C—C12C	113.26 (14)
C13A—C12A—C11A	122.4 (3)	C5C—C11C—H11C	104.9
C13X—C12A—C11A	126.8 (8)	C10C—C11C—H11C	104.9
C15X—C12A—C11A	123.0 (9)	C12C—C11C—H11C	104.9
C15A—C12A—C11A	124.8 (3)	C13C—C12C—C15C	111.74 (18)
C13A—S1A—C14A	92.9 (3)	C13C—C12C—C11C	124.78 (17)
C12A—C13A—S1A	112.0 (4)	C15C—C12C—C11C	123.30 (16)
C12A—C13A—H13A	124.0	C12C—C13C—S1C	112.38 (16)
S1A—C13A—H13A	124.0	C12C—C13C—H13D	123.8
C15A—C14A—S1A	110.8 (4)	S1C—C13C—H13D	123.8
C15A—C14A—H14A	124.6	C15C—C14C—S1C	103.73 (12)
S1A—C14A—H14A	124.6	C15C—C14C—H14D	128.1
C14A—C15A—C12A	112.1 (5)	S1C—C14C—H14D	128.1
C14A—C15A—H15A	124.0	C12C—C15C—C14C	114.57 (17)
C12A—C15A—H15A	124.0	C12C—C15C—H15D	122.7
C13X—S1X—C14X	89.2 (8)	C14C—C15C—H15D	122.7
C12A—C13X—S1X	114.3 (13)	C2C—C16C—H16G	109.5
C12A—C13X—H13B	122.8	C2C—C16C—H16H	109.5
S1X—C13X—H13B	122.8	H16G—C16C—H16H	109.5
C15X—C14X—S1X	113.4 (13)	C2C—C16C—H16I	109.5
C15X—C14X—H14B	123.3	H16G—C16C—H16I	109.5
S1X—C14X—H14B	123.3	H16H—C16C—H16I	109.5
C14X—C15X—C12A	112.8 (16)	C7C—C17C—H17G	109.5
C14X—C15X—H15B	123.6	C7C—C17C—H17H	109.5
C12A—C15X—H15B	123.6	H17G—C17C—H17H	109.5
C2A—C16A—H16A	109.5	C7C—C17C—H17I	109.5
C2A—C16A—H16B	109.5	H17G—C17C—H17I	109.5
H16A—C16A—H16B	109.5	H17H—C17C—H17I	109.5
C2A—C16A—H16C	109.5	C14D—S1D—C13D	97.20 (11)
H16A—C16A—H16C	109.5	C2D—O1D—C1D	122.12 (15)
H16B—C16A—H16C	109.5	C4D—O2D—H2OD	109.5
C7A—C17A—H17A	109.5	C7D—O4D—C6D	122.16 (15)
C7A—C17A—H17B	109.5	C9D—O5D—H5OD	109.5
H17A—C17A—H17B	109.5	O3D—C1D—O1D	114.79 (16)
C7A—C17A—H17C	109.5	O3D—C1D—C5D	126.83 (17)
H17A—C17A—H17C	109.5	O1D—C1D—C5D	118.37 (15)
H17B—C17A—H17C	109.5	C3D—C2D—O1D	121.08 (16)
C14B—S1B—C13B	92.19 (11)	C3D—C2D—C16D	126.60 (17)
C2B—O1B—C1B	122.25 (15)	O1D—C2D—C16D	112.31 (16)
C4B—O2B—H2OB	109.5	C2D—C3D—C4D	119.01 (16)
C7B—O4B—C6B	121.86 (15)	C2D—C3D—H3DA	120.5
C9B—O5B—H5OB	109.5	C4D—C3D—H3DA	120.5
O3B—C1B—O1B	114.30 (16)	O2D—C4D—C5D	118.40 (16)
O3B—C1B—C5B	126.67 (17)	O2D—C4D—C3D	120.41 (16)
O1B—C1B—C5B	119.03 (16)	C5D—C4D—C3D	121.19 (16)
C3B—C2B—O1B	120.65 (16)	C4D—C5D—C1D	118.19 (15)
C3B—C2B—C16B	127.19 (17)	C4D—C5D—C11D	119.88 (15)
O1B—C2B—C16B	112.15 (17)	C1D—C5D—C11D	121.72 (15)
C2B—C3B—C4B	119.05 (17)	O6D—C6D—O4D	113.86 (16)
C2B—C3B—H3BA	120.5	O6D—C6D—C10D	126.49 (17)
C4B—C3B—H3BA	120.5	O4D—C6D—C10D	119.62 (16)
O2B—C4B—C5B	118.02 (16)	C8D—C7D—O4D	120.74 (17)
O2B—C4B—C3B	120.99 (16)	C8D—C7D—C17D	127.49 (19)
C5B—C4B—C3B	120.98 (17)	O4D—C7D—C17D	111.78 (17)
C4B—C5B—C1B	117.87 (16)	C7D—C8D—C9D	119.59 (17)
C4B—C5B—C11B	119.80 (16)	C7D—C8D—H8DA	120.2
C1B—C5B—C11B	121.34 (15)	C9D—C8D—H8DA	120.2
O6B—C6B—O4B	113.97 (16)	O5D—C9D—C10D	125.00 (17)
O6B—C6B—C10B	126.61 (17)	O5D—C9D—C8D	114.56 (16)
O4B—C6B—C10B	119.40 (16)	C10D—C9D—C8D	120.42 (16)
C8B—C7B—O4B	120.80 (17)	C9D—C10D—C6D	117.47 (16)
C8B—C7B—C17B	127.64 (18)	C9D—C10D—C11D	127.24 (16)
O4B—C7B—C17B	111.56 (17)	C6D—C10D—C11D	115.05 (15)
C7B—C8B—C9B	120.27 (18)	C10D—C11D—C5D	118.00 (14)
C7B—C8B—H8BA	119.9	C10D—C11D—C12D	113.80 (14)
C9B—C8B—H8BA	119.9	C5D—C11D—C12D	112.12 (14)
O5B—C9B—C10B	125.28 (17)	C10D—C11D—H11D	103.6
O5B—C9B—C8B	114.63 (17)	C5D—C11D—H11D	103.6
C10B—C9B—C8B	120.08 (17)	C12D—C11D—H11D	103.6
C9B—C10B—C6B	117.51 (16)	C13D—C12D—C15D	112.65 (17)
C9B—C10B—C11B	127.35 (16)	C13D—C12D—C11D	125.56 (16)
C6B—C10B—C11B	114.95 (15)	C15D—C12D—C11D	121.14 (16)
C10B—C11B—C12B	115.40 (14)	C12D—C13D—S1D	111.51 (15)
C10B—C11B—C5B	118.62 (14)	C12D—C13D—H13E	124.2
C12B—C11B—C5B	109.55 (14)	S1D—C13D—H13E	124.2
C10B—C11B—H11B	103.7	C15D—C14D—S1D	108.31 (13)
C12B—C11B—H11B	103.7	C15D—C14D—H14E	125.8
C5B—C11B—H11B	103.7	S1D—C14D—H14E	125.8
C13B—C12B—C15B	112.31 (19)	C12D—C15D—C14D	110.33 (17)
C13B—C12B—C11B	127.15 (18)	C12D—C15D—H15E	124.8
C15B—C12B—C11B	120.13 (17)	C14D—C15D—H15E	124.8
C12B—C13B—S1B	111.24 (17)	C2D—C16D—H16J	109.5
C12B—C13B—H13C	124.4	C2D—C16D—H16K	109.5
S1B—C13B—H13C	124.4	H16J—C16D—H16K	109.5
C15B—C14B—S1B	111.77 (19)	C2D—C16D—H16L	109.5
C15B—C14B—H14C	124.1	H16J—C16D—H16L	109.5
S1B—C14B—H14C	124.1	H16K—C16D—H16L	109.5
C14B—C15B—C12B	112.5 (2)	C7D—C17D—H17J	109.5
C14B—C15B—H15C	123.8	C7D—C17D—H17K	109.5
C12B—C15B—H15C	123.8	H17J—C17D—H17K	109.5
C2B—C16B—H16D	109.5	C7D—C17D—H17L	109.5
C2B—C16B—H16E	109.5	H17J—C17D—H17L	109.5
H16D—C16B—H16E	109.5	H17K—C17D—H17L	109.5
C2B—C16B—H16F	109.5		
			
C2A—O1A—C1A—O3A	−179.33 (17)	C10B—C11B—C12B—C15B	175.72 (16)
C2A—O1A—C1A—C5A	0.1 (3)	C5B—C11B—C12B—C15B	−47.3 (2)
C1A—O1A—C2A—C3A	−2.5 (3)	C15B—C12B—C13B—S1B	0.5 (2)
C1A—O1A—C2A—C16A	176.25 (18)	C11B—C12B—C13B—S1B	173.08 (15)
O1A—C2A—C3A—C4A	2.4 (3)	C14B—S1B—C13B—C12B	−0.51 (18)
C16A—C2A—C3A—C4A	−176.1 (2)	C13B—S1B—C14B—C15B	0.4 (2)
C2A—C3A—C4A—O2A	179.17 (19)	S1B—C14B—C15B—C12B	−0.2 (3)
C2A—C3A—C4A—C5A	−0.1 (3)	C13B—C12B—C15B—C14B	−0.2 (3)
O2A—C4A—C5A—C1A	178.68 (18)	C11B—C12B—C15B—C14B	−173.36 (19)
C3A—C4A—C5A—C1A	−2.1 (3)	C2C—O1C—C1C—O3C	176.51 (18)
O2A—C4A—C5A—C11A	−4.9 (3)	C2C—O1C—C1C—C5C	−4.1 (3)
C3A—C4A—C5A—C11A	174.27 (17)	C1C—O1C—C2C—C3C	2.3 (3)
O3A—C1A—C5A—C4A	−178.51 (19)	C1C—O1C—C2C—C16C	−178.7 (2)
O1A—C1A—C5A—C4A	2.1 (3)	O1C—C2C—C3C—C4C	1.0 (3)
O3A—C1A—C5A—C11A	4.8 (3)	C16C—C2C—C3C—C4C	−177.7 (2)
O1A—C1A—C5A—C11A	−174.60 (15)	C2C—C3C—C4C—O2C	177.2 (2)
C7A—O4A—C6A—O6A	178.57 (16)	C2C—C3C—C4C—C5C	−2.6 (3)
C7A—O4A—C6A—C10A	−1.4 (2)	O2C—C4C—C5C—C1C	−178.96 (17)
C6A—O4A—C7A—C8A	1.2 (3)	C3C—C4C—C5C—C1C	0.8 (3)
C6A—O4A—C7A—C17A	−178.65 (16)	O2C—C4C—C5C—C11C	0.3 (3)
O4A—C7A—C8A—C9A	1.0 (3)	C3C—C4C—C5C—C11C	−179.90 (17)
C17A—C7A—C8A—C9A	−179.15 (19)	O3C—C1C—C5C—C4C	−178.29 (18)
C7A—C8A—C9A—O5A	176.43 (17)	O1C—C1C—C5C—C4C	2.4 (3)
C7A—C8A—C9A—C10A	−3.2 (3)	O3C—C1C—C5C—C11C	2.4 (3)
O5A—C9A—C10A—C6A	−176.60 (16)	O1C—C1C—C5C—C11C	−176.84 (16)
C8A—C9A—C10A—C6A	3.0 (2)	C7C—O4C—C6C—O6C	−176.05 (17)
O5A—C9A—C10A—C11A	2.4 (3)	C7C—O4C—C6C—C10C	3.1 (3)
C8A—C9A—C10A—C11A	−178.01 (16)	C6C—O4C—C7C—C8C	−3.1 (3)
O6A—C6A—C10A—C9A	179.30 (17)	C6C—O4C—C7C—C17C	176.25 (18)
O4A—C6A—C10A—C9A	−0.8 (2)	O4C—C7C—C8C—C9C	0.4 (3)
O6A—C6A—C10A—C11A	0.3 (3)	C17C—C7C—C8C—C9C	−178.8 (2)
O4A—C6A—C10A—C11A	−179.77 (15)	C7C—C8C—C9C—O5C	−178.6 (2)
C9A—C10A—C11A—C5A	98.78 (19)	C7C—C8C—C9C—C10C	2.2 (3)
C6A—C10A—C11A—C5A	−82.3 (2)	O5C—C9C—C10C—C6C	178.7 (2)
C9A—C10A—C11A—C12A	−128.12 (17)	C8C—C9C—C10C—C6C	−2.0 (3)
C6A—C10A—C11A—C12A	50.8 (2)	O5C—C9C—C10C—C11C	−4.4 (3)
C4A—C5A—C11A—C10A	72.6 (2)	C8C—C9C—C10C—C11C	174.84 (19)
C1A—C5A—C11A—C10A	−111.01 (18)	O6C—C6C—C10C—C9C	178.52 (19)
C4A—C5A—C11A—C12A	−61.2 (2)	O4C—C6C—C10C—C9C	−0.5 (3)
C1A—C5A—C11A—C12A	115.22 (17)	O6C—C6C—C10C—C11C	1.3 (3)
C10A—C11A—C12A—C13A	44.5 (6)	O4C—C6C—C10C—C11C	−177.67 (15)
C5A—C11A—C12A—C13A	178.8 (6)	C4C—C5C—C11C—C10C	98.34 (19)
C10A—C11A—C12A—C13X	−137.5 (19)	C1C—C5C—C11C—C10C	−82.4 (2)
C5A—C11A—C12A—C13X	−3.3 (19)	C4C—C5C—C11C—C12C	−129.79 (17)
C10A—C11A—C12A—C15X	48 (2)	C1C—C5C—C11C—C12C	49.5 (2)
C5A—C11A—C12A—C15X	−178 (2)	C9C—C10C—C11C—C5C	76.9 (2)
C10A—C11A—C12A—C15A	−144.5 (6)	C6C—C10C—C11C—C5C	−106.18 (18)
C5A—C11A—C12A—C15A	−10.3 (6)	C9C—C10C—C11C—C12C	−55.2 (3)
C13X—C12A—C13A—S1A	−7.0 (18)	C6C—C10C—C11C—C12C	121.73 (17)
C15X—C12A—C13A—S1A	67 (27)	C5C—C11C—C12C—C13C	−145.76 (18)
C15A—C12A—C13A—S1A	−0.8 (10)	C10C—C11C—C12C—C13C	−13.5 (3)
C11A—C12A—C13A—S1A	171.2 (4)	C5C—C11C—C12C—C15C	39.5 (2)
C14A—S1A—C13A—C12A	0.8 (7)	C10C—C11C—C12C—C15C	171.75 (17)
C13A—S1A—C14A—C15A	−0.6 (7)	C15C—C12C—C13C—S1C	0.2 (2)
S1A—C14A—C15A—C12A	0.2 (9)	C11C—C12C—C13C—S1C	−175.07 (14)
C13A—C12A—C15A—C14A	0.3 (11)	C14C—S1C—C13C—C12C	0.83 (16)
C13X—C12A—C15A—C14A	77 (10)	C13C—S1C—C14C—C15C	−1.43 (14)
C15X—C12A—C15A—C14A	−3(2)	C13C—C12C—C15C—C14C	−1.4 (2)
C11A—C12A—C15A—C14A	−171.4 (4)	C11C—C12C—C15C—C14C	173.99 (15)
C13A—C12A—C13X—S1X	5(3)	S1C—C14C—C15C—C12C	1.81 (19)
C15X—C12A—C13X—S1X	2(3)	C2D—O1D—C1D—O3D	179.64 (18)
C15A—C12A—C13X—S1X	−101 (11)	C2D—O1D—C1D—C5D	−0.4 (3)
C11A—C12A—C13X—S1X	−173.3 (9)	C1D—O1D—C2D—C3D	−0.7 (3)
C14X—S1X—C13X—C12A	−4(2)	C1D—O1D—C2D—C16D	−179.59 (18)
C13X—S1X—C14X—C15X	4(3)	O1D—C2D—C3D—C4D	0.2 (3)
S1X—C14X—C15X—C12A	−4(4)	C16D—C2D—C3D—C4D	178.9 (2)
C13A—C12A—C15X—C14X	−105 (29)	C2D—C3D—C4D—O2D	−179.47 (18)
C13X—C12A—C15X—C14X	2(4)	C2D—C3D—C4D—C5D	1.4 (3)
C15A—C12A—C15X—C14X	8(4)	O2D—C4D—C5D—C1D	178.42 (16)
C11A—C12A—C15X—C14X	177 (2)	C3D—C4D—C5D—C1D	−2.4 (3)
C2B—O1B—C1B—O3B	176.04 (17)	O2D—C4D—C5D—C11D	3.8 (2)
C2B—O1B—C1B—C5B	−3.3 (3)	C3D—C4D—C5D—C11D	−177.09 (16)
C1B—O1B—C2B—C3B	0.5 (3)	O3D—C1D—C5D—C4D	−178.13 (19)
C1B—O1B—C2B—C16B	−178.29 (16)	O1D—C1D—C5D—C4D	1.9 (3)
O1B—C2B—C3B—C4B	0.5 (3)	O3D—C1D—C5D—C11D	−3.6 (3)
C16B—C2B—C3B—C4B	179.04 (18)	O1D—C1D—C5D—C11D	176.47 (15)
C2B—C3B—C4B—O2B	−177.23 (18)	C7D—O4D—C6D—O6D	178.33 (17)
C2B—C3B—C4B—C5B	1.5 (3)	C7D—O4D—C6D—C10D	−0.2 (3)
O2B—C4B—C5B—C1B	174.54 (17)	C6D—O4D—C7D—C8D	−0.1 (3)
C3B—C4B—C5B—C1B	−4.2 (3)	C6D—O4D—C7D—C17D	−179.85 (16)
O2B—C4B—C5B—C11B	5.8 (3)	O4D—C7D—C8D—C9D	0.0 (3)
C3B—C4B—C5B—C11B	−172.99 (16)	C17D—C7D—C8D—C9D	179.71 (19)
O3B—C1B—C5B—C4B	−174.19 (19)	C7D—C8D—C9D—O5D	−178.09 (18)
O1B—C1B—C5B—C4B	5.1 (3)	C7D—C8D—C9D—C10D	0.4 (3)
O3B—C1B—C5B—C11B	−5.6 (3)	O5D—C9D—C10D—C6D	177.68 (18)
O1B—C1B—C5B—C11B	173.65 (15)	C8D—C9D—C10D—C6D	−0.6 (3)
C7B—O4B—C6B—O6B	176.13 (18)	O5D—C9D—C10D—C11D	3.5 (3)
C7B—O4B—C6B—C10B	−2.6 (3)	C8D—C9D—C10D—C11D	−174.79 (17)
C6B—O4B—C7B—C8B	0.8 (3)	O6D—C6D—C10D—C9D	−177.77 (19)
C6B—O4B—C7B—C17B	−178.62 (17)	O4D—C6D—C10D—C9D	0.5 (2)
O4B—C7B—C8B—C9B	0.4 (3)	O6D—C6D—C10D—C11D	−2.9 (3)
C17B—C7B—C8B—C9B	179.69 (19)	O4D—C6D—C10D—C11D	175.39 (15)
C7B—C8B—C9B—O5B	−178.79 (19)	C9D—C10D—C11D—C5D	−71.9 (2)
C7B—C8B—C9B—C10B	0.3 (3)	C6D—C10D—C11D—C5D	113.78 (17)
O5B—C9B—C10B—C6B	176.94 (18)	C9D—C10D—C11D—C12D	62.5 (2)
C8B—C9B—C10B—C6B	−2.1 (3)	C6D—C10D—C11D—C12D	−111.76 (17)
O5B—C9B—C10B—C11B	2.2 (3)	C4D—C5D—C11D—C10D	−108.44 (19)
C8B—C9B—C10B—C11B	−176.84 (17)	C1D—C5D—C11D—C10D	77.1 (2)
O6B—C6B—C10B—C9B	−175.38 (19)	C4D—C5D—C11D—C12D	116.38 (18)
O4B—C6B—C10B—C9B	3.2 (3)	C1D—C5D—C11D—C12D	−58.1 (2)
O6B—C6B—C10B—C11B	0.0 (3)	C10D—C11D—C12D—C13D	3.4 (3)
O4B—C6B—C10B—C11B	178.59 (16)	C5D—C11D—C12D—C13D	140.57 (19)
C9B—C10B—C11B—C12B	60.5 (2)	C10D—C11D—C12D—C15D	173.48 (17)
C6B—C10B—C11B—C12B	−114.43 (18)	C5D—C11D—C12D—C15D	−49.4 (2)
C9B—C10B—C11B—C5B	−72.5 (2)	C15D—C12D—C13D—S1D	−0.7 (2)
C6B—C10B—C11B—C5B	112.64 (18)	C11D—C12D—C13D—S1D	170.07 (14)
C4B—C5B—C11B—C10B	−115.59 (19)	C14D—S1D—C13D—C12D	0.55 (18)
C1B—C5B—C11B—C10B	76.1 (2)	C13D—S1D—C14D—C15D	−0.22 (16)
C4B—C5B—C11B—C12B	108.99 (18)	C13D—C12D—C15D—C14D	0.5 (2)
C1B—C5B—C11B—C12B	−59.4 (2)	C11D—C12D—C15D—C14D	−170.69 (16)
C10B—C11B—C12B—C13B	3.6 (3)	S1D—C14D—C15D—C12D	−0.1 (2)
C5B—C11B—C12B—C13B	140.63 (19)		

### Hydrogen-bond geometry (Å, °)

**Table:**

*D*—H···*A*	*D*—H	H···*A*	*D*···*A*	*D*—H···*A*
O2A—H2OA···O6A	0.82	1.78	2.562 (2)	159
O5A—H5OA···O6D	0.82	1.84	2.634 (2)	164
O2B—H2OB···O6C^i^	0.82	1.85	2.631 (2)	158
O5B—H5OB···O3B	0.82	1.71	2.529 (2)	171
O2C—H2OC···O6B^ii^	0.82	1.84	2.663 (2)	175
O5C—H5OC···O3C	0.82	1.76	2.549 (2)	163
O2D—H2OD···O3A	0.82	1.82	2.6205 (19)	166
O5D—H5OD···O3D	0.82	1.81	2.546 (2)	148
C3A—H3AA···O3C	0.93	2.45	3.306 (3)	154
C8A—H8AA···O6D	0.93	2.38	3.072 (2)	131
C3B—H3BA···O6A^iii^	0.93	2.51	3.258 (2)	138
C3C—H3CA···O6B^ii^	0.93	2.52	3.193 (3)	130
C11A—H11A···O3A	0.98	2.34	2.871 (2)	113
C11A—H11A···O5A	0.98	2.30	2.805 (2)	111
C11B—H11B···O2B	0.98	2.22	2.768 (2)	114
C11B—H11B···O6B	0.98	2.27	2.835 (2)	115
C11C—H11C···O2C	0.98	2.35	2.826 (2)	109
C11C—H11C···O6C	0.98	2.36	2.875 (2)	112
C11D—H11D···O2D	0.98	2.23	2.777 (2)	114
C11D—H11D···O6D	0.98	2.27	2.833 (2)	115
C13A—H13A···O4C^iv^	0.93	2.39	3.278 (8)	159
C13B—H13C···O1A^v^	0.93	2.51	3.311 (3)	145
C13D—H13E···O1B^vi^	0.93	2.59	3.366 (2)	142
C14A—H14A···O3C^vii^	0.93	2.33	3.183 (4)	152
C14C—H14D···O1C^vii^	0.93	2.52	3.377 (2)	154
C16B—H16D···O6A^iii^	0.96	2.58	3.382 (3)	141

Symmetry codes: (i) *x*, *y*+1, *z*+1; (ii) *x*, *y*−1, *z*−1; (iii) −*x*, −*y*+2, *z*+1/2; (iv) −*x*, −*y*+1, *z*+1/2; (v) −*x*+1/2, *y*, *z*+1/2; (vi) *x*, *y*−1, *z*; (vii) *x*, *y*+1, *z*.

## References

[bb1] Abe, I., Oguro, S., Utsumi, Y., Sano, Y. & Noguchi, H. (2005). *J. Am. Chem. Soc.* **127**, 12709–12716. 10.1021/ja053945v 16144421

[bb2] Allen, F. H., Kennard, O., Watson, D. G., Brammer, L., Orpen, A. G. & Taylor, R. (1987). *J. Chem. Soc. Perkin Trans. 2*, pp. S1–19.

[bb3] Bentley, R. (1999). *J. Chem. Educ.* **76**, 41–47.

[bb4] Bruker (2009). *APEX2*, *SAINT* and *SADABS* Bruker AXS Inc., Madison, Wisconsin, USA.

[bb5] Cosier, J. & Glazer, A. M. (1986). *J. Appl. Cryst.* **19**, 105–107.

[bb6] Eckermann, C., Schroder, G., Eckermann, S., Strack, D., Schmidt, J., Schneider, B. & Schroder, J. (2003). *Phytochemistry*, **62**, 271–286. 10.1016/s0031-9422(02)00554-x 12620338

[bb7] Fang, Z., Liao, P.-C., Yang, Y.-L., Yang, F.-L., Chen, Y.-L., Lam, Y., Hua, K.-F. & Wu, S.-H. (2010). *J. Med. Chem.* **53**, 7967–7978. 10.1021/jm100619x 20964408

[bb8] Flack, H. D. (1983). *Acta Cryst.* A**39**, 876–881.

[bb9] Lee, Y. S., Kima, S. N., Lee, Y. S., Lee, J. Y., Lee, C.-K., Kim, H. S. & Park, H. (2000). *Arch. Pharm. Pharm. Med. Chem.* **333**, 319–322. 10.1002/1521-4184(200010)333:10<319::aid-ardp319>3.0.co;2-a 11092133

[bb10] Sheldrick, G. M. (2008). *Acta Cryst.* A**64**, 112–122. 10.1107/S0108767307043930 18156677

[bb11] Shyamsunder, C. & Hermann, H. (1999). *Chem. Abstr.* **130**, 338018h.

[bb12] Spek, A. L. (2009). *Acta Cryst.* D**65**, 148–155. 10.1107/S090744490804362X PMC2631630 19171970

